# Liver fat: a relevant target for dietary intervention? Summary of a Unilever workshop

**DOI:** 10.1017/jns.2017.13

**Published:** 2017-05-08

**Authors:** Harry P. F. Peters, Patrick Schrauwen, Petra Verhoef, Christopher D. Byrne, David J. Mela, Andreas F. H. Pfeiffer, Ulf Risérus, Frits R. Rosendaal, Vera Schrauwen-Hinderling

**Affiliations:** 1 Unilever R&D Vlaardingen, Olivier van Noortlaan 120, Vlaardingen, The Netherlands; 2 Department of Human Biology, NUTRIM School for Nutrition and Translational Research in Metabolism, Maastricht University Medical Center, Maastricht, The Netherlands; 3 Nutrition and Metabolism, Faculty of Medicine, University of Southampton & Southampton National Institute for Health Research Biomedical Research Centre, University Hospital Southampton, Southampton, UK; 4 Department of Endocrinology, Diabetes and Nutrition, Charité Universitätsmedizin Berlin, Campus Benjamin Franklin, Berlin; 5 Department of Clinical Nutrition, German Institute of Human Nutrition, Potsdam and German Center for Diabetes Research, DZD, Neuherberg, Germany; 6 Department of Public Health and Caring Sciences, Clinical Nutrition and Metabolism Unit, Uppsala University, Sweden; 7 Department of Clinical Epidemiology, Leiden University Medical Center, Leiden, The Netherlands; 8 Department of Radiology, NUTRIM School for Nutrition and Translational Research in Metabolism, Maastricht University Medical Center, Maastricht, The Netherlands

**Keywords:** Liver fat, Diet, Type 2 diabetes, Cardiovascular disease, DNL, *de novo* lipogenesis, GI, glycaemic index, MRS, magnetic resonance spectroscopy, NAFLD, non-alcoholic fatty liver disease, NASH, non-alcoholic steatohepatitis, PET, positron emission tomography, PNPLA3, patatin-like phospholipase domain-containing protein 3 gene, T2DM, type 2 diabetes mellitus

## Abstract

Currently it is estimated that about 1 billion people globally have non-alcoholic fatty liver disease (NAFLD), a condition in which liver fat exceeds 5 % of liver weight in the absence of significant alcohol intake. Due to the central role of the liver in metabolism, the prevalence of NAFLD is increasing in parallel with the prevalence of obesity, insulin resistance and other risk factors of metabolic diseases. However, the contribution of liver fat to the risk of type 2 diabetes mellitus and CVD, relative to other ectopic fat depots and to other risk markers, is unclear. Various studies have suggested that the accumulation of liver fat can be reduced or prevented via dietary changes. However, the amount of liver fat reduction that would be physiologically relevant, and the timeframes and dose–effect relationships for achieving this through different diet-based approaches, are unclear. Also, it is still uncertain whether the changes in liver fat *per se* or the associated metabolic changes are relevant. Furthermore, the methods available to measure liver fat, or even individual fatty acids, differ in sensitivity and reliability. The present report summarises key messages of presentations from different experts and related discussions from a workshop intended to capture current views and research gaps relating to the points above.

## Background

The liver plays a central role in the metabolic fluxes within the human body, in particular in the postprandial state. Therefore, it may play a crucial role in the relationships of diet with cardiometabolic health. When fat accumulates in the liver, it can adversely affect the functioning of the liver itself, as well as causing extrahepatic metabolic disturbances. Obesity and elevated postprandial glycaemic and lipidaemic responses are associated with ectopic fat accumulation in general, and possibly also with liver fat accumulation.

When liver fat exceeds 5 % of liver weight in the absence of significant alcohol intake, or other established risk factors for liver fat accumulation, it is called non-alcoholic fatty liver disease (NAFLD^(^^1^^)^). In addition to a higher risk of liver disease-related mortality and morbidity, NAFLD is associated with increased risk of type 2 diabetes mellitus (T2DM) and CVD, though the causality of this link is still debated^(^^2^^–^^6^^)^. Currently it is estimated that about 1 billion people globally have NAFLD^(^^7^^)^, and the prevalence is increasing in parallel with the prevalence of obesity, insulin resistance and other metabolic syndrome parameters^(^^1^^,^^8^^)^.

**Fig. 1.**
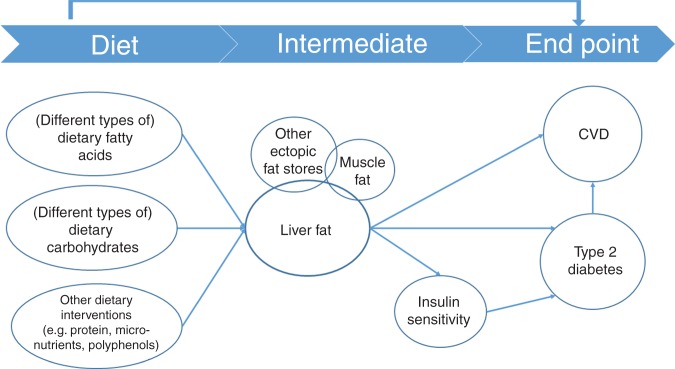
The potential relationship between diet, intermediates like liver fat or insulin sensitivity, and end points like type 2 diabetes mellitus and CVD.

Due to the central role of the liver in metabolism, reducing liver fat content is potentially a key target in the prevention and treatment of metabolic diseases. However, liver fat is only one of many possible physiological targets, and diet and lifestyle changes aimed at reducing liver fat often beneficially affect other metabolic parameters. Also, the contribution of hepatic fat to risk of T2DM and CVD, relative to other ectopic fat depots (e.g. in muscles) and to other risk markers (e.g. lipidaemia, insulin resistance) needs further clarification. The possible relationships among diet, intermediates like liver fat or insulin sensitivity, and disease end points like T2DM and CVD are visualised in Fig. 1.

With the availability of improved non-invasive techniques for measuring hepatic fat content, such as MRI and magnetic resonance spectroscopy (MRS), the role of fatty liver in health and disease is being studied more intensely and reliably^(^^9^^–^^11^^)^. Various studies have suggested that the accumulation of liver fat can be reduced or prevented via dietary changes^(^^12^^–^^16^^)^. However, the amount of liver fat reduction that would be physiologically relevant, and the timeframes and dose–effect relationships for achieving this through different diet-based approaches are still unclear.

To capture current views and research gaps relating to these points, Unilever organised a workshop with external experts in NAFLD and metabolic disease on 3 November 2015 in Vlaardingen, The Netherlands. The invited academic and Unilever scientists together covered a broad range of expertise in epidemiology, hepatology, endocrinology, metabolism, physiology, imaging and nutrition. The ultimate objective of the meeting was to understand the importance of liver fat as a target, its link with cardiometabolic diseases, and the potential strategies for lowering liver fat levels by diet.

The five key questions to be addressed during the workshop were:

Q1. Is fatty liver (accepted as) an independent risk factor for type 2 diabetes mellitus?

Q2. Is fatty liver (accepted as) an independent risk factor for cardiovascular disease?

Q3. Can the amount of liver fat be measured non-invasively, reliably and validly?

Q4. What amount of liver fat reduction/prevention is considered relevant in view of health and disease prevention?

Q5. Which dietary approaches are effective in reducing or preventing liver fat/fatty liver?

Each academic expert addressed one or more of these questions in his or her presentation, followed by discussions in subgroups and a plenary discussion to obtain a consensus on answers to these questions. This report summarises the key messages of the presentations, and concludes with the overall view of the speakers and other participants at the meeting.

## Fatty liver: the food industry perspective

Dr David Mela opened the workshop by noting that most large global food manufacturers have stated goals for monitoring and improving the nutritional quality of their products and portfolio. For example, under the Unilever Sustainable Living Plan, Unilever has set out a range of specific nutrition targets (i.e. lowering energy, sugar, salt, *trans*-fat and saturated fat content) to improve the health and well-being of consumers, including reduction in risk of cardiometabolic diseases. Given the increasing prevalence of fatty liver and its suggested association with risk of T2DM and CVD, there is a growing interest in whether specific (manufactured) foods or food ingredients can contribute to reducing risk of fatty liver and its consequences.

He emphasised that the food industry's approaches to the delivery of health benefits differ in important ways from pharmaceutical approaches. Diet will always be seen as one contributor to a healthier lifestyle and reduced disease risk, and individual foods cannot be claimed to treat, prevent or cure cardiometabolic disease in non-diseased populations. Individual foods can have beneficial physiological effects that may be linked to a potential for reducing cardiometabolic risk in the general population, and can have a wide reach in terms of affordability, numbers of people consuming these foods and consumption occasions.

Although fatty liver is emerging as a potential target of interest in the scientific community, industrial relevance for the development of products aimed at reducing liver fat depends on the sensitivity to dietary changes and substantiation of specific benefits of reducing fatty liver. It first needs to be established whether fatty liver is (1) a widely accepted physiological marker that reflects a ‘physiological benefit’ relevant for the general population, and (2) amenable to measurable beneficial dietary effects that can be a basis for meaningful consumer communication (claims), and (3) that these aspects are recognised and endorsed by experts including regulatory authorities. This workshop is a starting point to define where the science is now, where the gaps in knowledge are, and which additional academic and industrial research is needed to underpin the suggested health relevance of preventing and reducing fatty liver, and its implications in terms of disease risk.

## Ectopic fat and its consequences – who, where, what?: the physiologist's perspective

Professor Patrick Schrauwen proceeded to illustrate obesity as the major risk factor for the development of T2DM. In obesity, excessive fat is stored in white adipose tissue, but can also accumulate in ectopic sites like liver, muscle, heart and pancreas (e.g. Szczepaniak *et al.*^(^^17^^)^). Ectopic fat accumulation can exert lipotoxic effects leading to cellular dysfunction (e.g. impaired cardiac function^(^^17^^)^) and is responsible for metabolic complications of obesity, like insulin resistance, impaired insulin secretion and impaired glucose uptake in various tissues (e.g. Jacob *et al.*^(^^8^^)^ and Thamer *et al.*^(^^18^^)^). Therefore, ectopic fat accumulation largely explains the link between obesity and diabetes.

Having said that, it has previously been shown by Szczepaniak *et al*.^(^^11^^)^ that a significant correlation between BMI and liver fat content exists; however, this correlation is not very strong and liver fat accumulation is not limited to obese subjects. Thus, lean subjects with high amounts of liver fat can be identified and, vice versa, obese subjects with liver fat levels below the 5 % threshold. This makes the selection of the target population for interventions aiming at liver fat reduction not immediately obvious.

Why fat accumulates in metabolic active tissues is not completely understood, but elevated circulating NEFA levels have been suggested to underlie ectopic fat accumulation^(^^19^^)^. Increasing the level of circulating NEFA *in vivo*, for example by performing acute exercise in the fasted state^(^^20^^)^, leads to an increase in cardiac and hepatic lipid content^(^^21^^)^. However, regular exercise training, leading to an improvement in oxidative capacity, lowers fat accumulation in liver and the heart^(^^22^^)^. Indeed, numerous other studies have addressed the positive role of exercise and physical activity for reducing liver fat and other ectopic fat stores^(^^16^^,^^23^^,^^24^^)^.

Although in general a reduction in ectopic fat is associated with beneficial health effects, the relationship between fat accumulation and lipotoxicity is not always straightforward and not always present in all tissues. For example, supplementation with resveratrol may increase muscle fat, but at the same time may improve metabolic health, including a decrease in liver fat^(^^25^^)^. Another intriguing example is that endurance-trained athletes have high amounts of fat stored in the muscle (intramyocellular lipid), yet their insulin sensitivity is not compromised. Furthermore, long-term training increases rather than decreases muscle fat content^(^^26^^)^. This has been called ‘the athlete's paradox’^(^^27^^)^. Although this paradox has not been completely solved, it is suggested that increased channelling of fatty acids toward storage in the form of inert TAG in skeletal muscle is associated with an improved metabolic profile, and that this capacity is blunted in T2DM. As a result, intermediates of fatty acid metabolism, such as diacylglycerol and ceramides, may accumulate and be responsible for the lipotoxic effects in skeletal muscle. A high capacity to channel fatty acids toward intramyocellular lipid storage may therefore protect against lipid-induced insulin resistance. Importantly, recent evidence suggests that also in the liver, fat accumulation is not *per se* always detrimental^(^^28^^)^. This may partly depend on factors like tissue oxidative capacity, lipid droplet dynamics and lipid turnover^(^^29^^)^. It remains to be elucidated which factors determine whether a high fat content in liver or muscle is either detrimental or protective. Non-invasive measurement of the different fats and fat intermediates in liver and other ectopic fat stores will help to unravel this.

## Liver fat: the epidemiologist's perspective

Professor Frits Rosendaal elaborated on the definition and prevalence of NAFLD. NAFLD is regarded as a container term^(^^1^^)^ as it encompasses various subtypes. Two major subtypes are (1) non-alcoholic fatty liver (also termed simple steatosis), the non-progressive form of NAFLD that rarely develops into cirrhosis, and (2) non-alcoholic steatohepatitis (NASH), the progressive form of NAFLD that can lead to cirrhosis, hepatocellular carcinoma and liver-related mortality. Whether NAFLD really leads to NASH is under debate, with distinct pathogenic pathways claimed for hepatic steatosis and NASH, but it has been estimated that about 30 % of people with NAFLD eventually develop NASH^(^^30^^)^.

In the industrialised world it is typically estimated that 20–30 % of adults have NAFLD, but the prevalence figures range from 6 to 51 %, depending on the assessment method, definition, country, region and ethnicity^(^^7^^,^^31^^)^. Prevalence also depends on the risk groups. In general, NAFLD prevalence is higher at older age, lower in people of African descent, not clearly different between sexes, about 70 % in T2DM, at least 50 % in overweight and over 70 % in obese people^(^^32^^–^^37^^)^. Even in children a mean prevalence of 9·6 % is reported, with even higher numbers in 15- to 17-year-olds^(^^38^^)^.

NAFLD is associated with increased death rates, particularly from CVD (IHD, ischaemic stroke) and liver disease (cirrhosis)^(^^1^^,^^39^^)^. Observational studies provide evidence of a strong association between NAFLD and subclinical manifestation of atherosclerosis (e.g. Targher *et al.*^(^^5^^)^). Nevertheless, it remains controversial whether fatty liver causes atherosclerosis and CVD, even though some underlying mechanisms for the causal relationship, such as inflammation and impaired haemostasis, seem plausible^(^^40^^)^. Rosendaal specifically referred to a clinical review^(^^41^^)^ that concluded ‘There is no convincing evidence that NAFLD independently increases a patient's CVD risk’.

Rosendaal went on to state that instead more consensus can be found for the view that NAFLD is a risk factor for insulin resistance and diabetes. The majority of studies to date suggest that liver fat accumulation leads to liver insulin resistance, via impaired insulin signalling. This reduced insulin sensitivity in turn is associated with the progression of NAFLD, although the causality of this (circular) relationship is under debate. Despite absence of proof of causality, subjects with NAFLD have a high risk of developing T2DM^(^^4^^,^^42^^,^^43^^)^, possibly reflecting co-association of NAFLD with other risk factors for T2DM, in particular obesity and insulin resistance. A recent meta-analytical study quantified that patients with NAFLD have a two-fold increased risk of T2DM^(^^3^^)^.

Rosendaal returned to the question of whether fatty liver is (accepted as) a risk factor for CVD or T2DM. He discussed whether it is essential that a relationship is defined as ‘independent’ of other risk factors. Liver fat does not stand on its own, as it is related to other types of ectopic fat and risk factors, and there could also be an interplay between NAFLD and the metabolic syndrome. It is therefore better to talk about ‘causal risk factor’ (i.e. part of the causal pathway) instead of ‘independent’, which is a multi-interpretable word usually intended to imply the absence of confounding. Causality is defined as when an event occurs in the presence, but would not have occurred in the absence of a certain factor in the past. In that situation, one can assume a causal link between the factor and the following event. The strongest evidence therefore is experimental manipulation of the factor under question, such as to test a drug that only affects liver fat and no other metabolic risk factors directly, to understand the mechanism behind the relationship between fatty liver and metabolic diseases. Alternatives are observational studies, which, however, will suffer from the confounding by related variables, such as excess fat elsewhere. An interesting pseudo-experimental method is to use Mendelian randomisation studies, i.e. focus on genetic variants that influence the likelihood of having NAFLD and to test whether these also are linked to the risk of CVD or T2DM. One attempt to test causality between fatty liver and metabolic factors was performed by Zhang *et al*.^(^^44^^)^ applying a Bayesian network approach to two large longitudinal cohorts. Generalised estimating equation analyses suggested that NAFLD was indeed a cause of T2DM, but the other way around appeared more likely. However, fatty liver was assessed by ultrasound, which has a low sensitivity, and the epidemiological analyses were limited to a specific group of urban Chinese subjects.

Currently several large cohorts are ongoing where liver fat is measured sensitively and reliably using MRI and MRS (for a discussion, see below) and subjects are followed for many years. One such large prospective cohort study is The Netherlands Epidemiology of Obesity (NEO) study, designed to investigate pathways that lead to obesity-related diseases such as T2DM. Study subjects have been phenotyped extensively (e.g. diet, insulin sensitivity and several ectopic fat depots, including liver fat) and results will be available in 2019^(^^45^^)^. These and other data will be used to model the expected health benefits of liver fat reduction.

## The state of the art and future prospects of measuring fatty liver non-invasively: the methodologist's perspective

Dr Vera Schrauwen-Hinderling explained that liver fat content can be determined by various means. The ‘gold standard’ for diagnosis of NAFLD used to be the liver biopsy, but it is only justified in severe liver disease and comes with many drawbacks, such as sampling error, cost and risk of complications^(^^46^^)^. Biopsy is still considered the ‘gold standard’ for establishing NASH^(^^46^^)^. Efforts are ongoing, including the use of MRS, to distinguish non-invasively between inflamed and non-inflamed fatty liver. Only then can the effect of the inflammatory state of the liver on subsequent disease risk be reliably assessed at a larger scale.

Liver enzymes are often used to screen for potentially elevated liver fat and these are indeed usually increased in NAFLD patients. However, a large proportion of NAFLD patients exhibit normal liver enzyme levels. In addition, combinations of plasma enzyme levels and anthropometrics are used to estimate liver fat, but so far none of these has high sensitivity and specificity^(^^47^^)^.

Ultrasound sonography is widely used in the clinical setting. The sensitivity of this method, however, is rather low and therefore only severe steatosis can be detected reliably. Furthermore, the use of ultrasound is difficult in subjects with high amounts of subcutaneous adipose tissue^(^^48^^)^. To assess small changes other methods are needed.

Other imaging methods are much more accurate: fat-selective and water-selective MRI or computer tomography can be used to determine liver fat content, with proton MRS (^1^H-MRS) being the most sensitive method^(^^49^^)^. MRS reliably detects fat quantities as low as 0·5 %, has excellent reproducibility and sensitivity, and is generally considered the most sensitive non-invasive method for detecting liver fat^(^^11^^,^^49^^)^. Due to the high accuracy and the absence of ionising radiation, this is also the method of choice to perform repeated measurements to determine small changes in liver fat due to interventions.

Further improvements in MRS techniques, such as high-quality motion correction and individual post-processing of single acquisitions, increases sensitivity even further together with signal gain due to increased number of averages, higher field strength and a large voxel size. Using such methodological improvements, even a small increase in liver fat after ingestion of a single high-fat meal could be visualised in healthy subjects^(^^9^^)^.

Usually, the lipid signals are normalised to the water signal and absolute concentrations are calculated based on assumptions of water content and relaxation kinetics. This may be problematic in conditions where hepatic water content may change, but this can be overcome by monitoring water content with MRI and external phantoms. Liver fat content (fat:water ratio) can also be measured by MRI (fat-selective and water-selective MRI) and although it shows a high correlation with MRS, MRI can give an overestimation at low fat fractions and is less sensitive than MRS^(^^50^^,^^51^^)^. MRI can also be used to measure liver volume. MRI can be of added value to MRS when it is expected that liver fat is not homogeneously distributed (e.g. in severe steatosis with focal fat accumulation).

MRS not only yields information about hepatic lipid content, but also on its composition, such as a saturation index, reflecting the relative abundance of SFA and unsaturated fatty acids. Haus *et al*.^(^^52^^)^, for example, showed that the saturation of hepatic fatty acids decreased after short-term exercise training. MR-based techniques, e.g. with two-dimensional spectroscopy, are under development that also allow comparison of levels of different specific unsaturated fatty acids in the liver.

Schrauwen-Hinderling further elaborated on the different metabolic sources of liver fat. Hepatic fat can be derived directly from a meal, from adipose tissue lipolysis or from *de novo* lipogenesis (DNL). The contribution of these different routes is not easy to determine in health and disease, and data in humans are currently limited to patients with fatty liver disease, scheduled for liver biopsies. Novel magnetic resonance-based and positron emission tomography (PET)-based techniques are emerging that enable the study of these pathways and the intervention effects in a broader population and in more detail. For example, using novel MRS techniques, stable isotope ^13^C-labelled fatty acids, in combination with a liquid meal, can be used for real-time dynamic tracking of dietary fatty acids to the liver^(^^53^^)^. These novel methods can complement currently available PET techniques, which use the radioactive fatty acid analogue ^18^fluorothia-6-heptadecanoic acid (^18^FTHA)^(^^54^^,^^55^^)^. The radioactive tracer used for PET is ingested orally and can be visualised in the liver, where it is trapped; therefore hepatic uptake of dietary fat can be quantified with PET. As DNL is also considered to contribute significantly to hepatic fat content, efforts are made to quantify DNL non-invasively. As DNL results in SFA, the level of fatty acid saturation is currently under discussion as an indicator of DNL, as well as the detection of ^13^C fat in liver in combination with ^13^C glucose intake.

## The role of fatty liver in insulin sensitivity and type 2 diabetes mellitus: the clinician's perspective

Professor Andreas Pfeiffer stated that liver fat content is clearly related to insulin sensitivity upon insulin infusion in normal subjects and people with T2DM, while basal insulin sensitivity shows little correlation. The closest relationship with liver fat is seen for hepatic insulin sensitivity and adipose tissue insulin sensitivity^(^^56^^,^^57^^)^. Liver fat is also negatively correlated with insulin clearance, both in non-diabetic subjects and T2DM patients^(^^58^^,^^59^^)^, and also negatively correlated with the suppression of endogenous glucose production^(^^59^^)^. An increase in incident T2DM is even observed with increased liver fat in ‘healthy obese phenotypes’^(^^60^^)^, where elevated liver fat appears to be a proxy for disturbed energy metabolism.

A recent study^(^^61^^)^ suggests that reduced ‘glucose effectiveness’, the insulin-independent component of glucose disposal comprising hepatic glucose uptake, is an important contributor to the risk of developing T2DM in a situation of increased liver fat. Others have shown that hepatic β-oxidation rates are increased in NAFLD and NASH compared with controls^(^^62^^)^ and this contributes to increased glucose production in the liver. However, glucose production in fatty liver disease appears to be increased from all three pathways, i.e. glycerol, gluconeogenesis and glycogenolysis as shown by elegant tracer studies^(^^63^^)^.

Pfeiffer highlighted that the majority of studies suggest that hepatic fat accumulation leads to insulin resistance, but he showed a few exceptions where high liver fat is dissociated from hepatic insulin resistance. One such dissociation is seen in familial hypobetalipoproteinaemia^(^^64^^)^. This suggests that liver fat content, at least in this case, may rather be a marker than a direct cause of hepatic insulin resistance. Another example is a recent study from Cuthbertson *et al*.^(^^65^^)^. While exercise reduced liver fat from 19·4 to 10·1 % together with a 4·9 kg weight loss, whole-body but not liver insulin resistance was improved. Gastric bypass or liver transplant studies may shed some light on causality and underlying mechanisms.

NAFLD is associated with hepatic and adipose tissue insulin resistance and the presence of NASH further impairs insulin sensitivity^(^^66^^)^. NASH is often associated with increased hepatic mitochondrial fatty acid oxidation and dysmorphic mitochondria. Inflammation is thought to support the induction of fibrogenesis and hepatic cirrhosis^(^^66^^)^ and also a role of endoplasmic reticulum stress is claimed in this respect^(^^67^^)^. Furthermore, there is some evidence that patients with NASH have a higher risk for developing T2DM than those with simple steatosis^(^^30^^)^.

Dr Pfeiffer continued his presentation by elaborating on the role of high carbohydrate intake. This promotes liver fat accumulation due to activation of the carbohydrate-responsive element-binding protein (ChREBP) and insulin-activated sterol regulatory element-binding transcription factor-1c (SREBP-1c) lipogenic pathways^(^^68^^)^.

Reduced liver fat accumulation is observed with low-glycaemic index (GI) foods^(^^69^^)^, which appears to relate to alterations of gastric inhibitory polypeptide (GIP) or glucagon-like peptide-1 (GLP-1)^(^^70^^)^. Nevertheless, GLP-1 agonists do not appear to specifically alter hepatic fat accumulation but rather act in the context of general metabolic improvement^(^^71^^)^. Reduced liver fat accumulation is also observed when carbohydrate absorption is delayed, e.g. by using the drug acarbose^(^^72^^)^ or by using the slowly absorbable sugar palatinose^(^^73^^)^.

There are no controlled studies directly demonstrating that reductions of liver fat reduce the incidence of T2DM, although this appears likely, considering diabetes prevention studies. There are several studies showing the effectiveness of dietary interventions on liver fat (e.g. Browning *et al.*^(^^74^^)^, Kruse *et al.*^(^^75^^)^ and Nowotny *et al.*^(^^76^^)^). However, since liver fat is quite variably associated with insulin resistance, Dr Pfeiffer concluded that there are probably no absolute numbers (threshold value) for a ‘preventive reduction’ of liver fat and the efficiency of different strategies needs to be investigated.

## Extrahepatic complications of non-alcoholic fatty liver disease – type 2 diabetes mellitus and CVD: the metabolic physician/diabetologist's perspective

Professor Christopher Byrne proceeded to illustrate the view that liver fat content exacerbates hepatic insulin resistance, predisposes to atherogenic dyslipidaemia and also increases the risk of developing T2DM and CVD^(^^3^^)^.

Byrne & Targher^(^^77^^)^ recently evaluated all studies using non-invasive imaging techniques (predominantly ultrasonography) that looked into associations between incident T2DM and incident and existing fatty liver. Nearly all studies have shown that NAFLD increases incident T2DM risk and that the risk, which varied from a 1·6- to a 5·5-fold increase, probably depends on the NAFLD severity. The wide variation in risk estimates might also reflect differences in the number and type of covariates adjusted for. Interestingly, a retrospective study of a Korean occupational cohort of 13 000 subjects studied at baseline and at 5-year follow-up showed that insulin resistance alone increased the risk for T2DM four-fold, fatty liver alone increased risk three-fold, while the combination increased the risk seven-fold. When these subjects were also obese, the fully adjusted OR increased to approximately 14^(^^78^^)^.

The relationship of NAFLD with risk of T2DM has been further corroborated by the results of a recent retrospective study that also assessed the impact of resolution of fatty liver over 5 years of follow-up on the risk of incident diabetes at 5-year follow-up^(^^79^^)^. These data showed that amelioration of fatty liver (on ultrasound examination) between baseline and follow-up examination attenuated the risk of incident type 2 diabetes at follow-up to the same risk as subjects who did not have fatty liver at baseline or follow-up examinations. Thus it is plausible that resolution or improvement in liver lipid metabolism modifies T2DM risk^(^^77^^)^. Nevertheless, more studies are needed to verify that improvement in NAFLD limits the risk of T2DM or improves glycaemic control in people with NAFLD who have developed T2DM.

While NAFLD increases the risk of developing T2DM, even more studies evaluated CVD risk, assessed either by fatal CVD accidents or by markers for subclinical CVD. A recent comprehensive meta-analysis^(^^80^^)^ of twenty-seven cross-sectional studies showed a strong association between NAFLD and various markers of subclinical CVD. These authors concluded that despite the evidence to support the independent association of NAFLD with subclinical atherosclerosis, there is still a need for future longitudinal studies to review this association to ascertain causality and include other ethnic populations. Byrne & Targher presented another analysis^(^^77^^)^ of seventeen observational studies assessing risk of CVD mortality and morbidity in patients with NAFLD (mostly diagnosed by ultrasound or biopsy) and most of these studies showed an increased risk. Several large cross-sectional population and hospital-based studies, involving patients with and without diabetes, also have consistently shown that the prevalence of clinical CVD is increased in patients with NAFLD (e.g. Stepanova & Younossi^(^^81^^)^ and Targher *et al.*^(^^82^^)^). CVD risk seems to be particularly increased in NASH, but is certainly also elevated in NAFLD^(^^83^^)^.

**Fig. 2.**
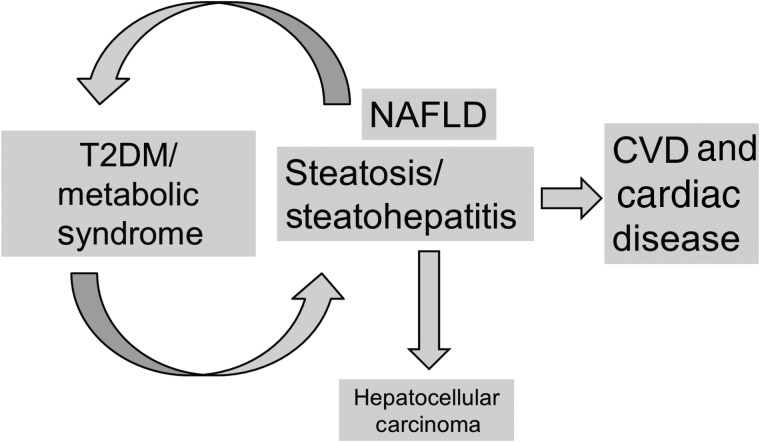
Type 2 diabetes mellitus (T2DM) and non-alcoholic fatty liver disease (NAFLD): a vicious cycle for CVD (design Christopher Byrne).

NAFLD appears not only to contribute to the development of atherosclerosis, but it also influences other structural and functional cardiac alterations, such as cardiac calcification in aortic and mitral valves^(^^84^^)^ or atrial fibrillation in T2DM patients^(^^85^^)^ and increases the risk of developing hypertension (e.g. Sung *et al.*^(^^86^^)^). Fig. 2 summarises the complex relationship between T2DM and NAFLD and its effect on CVD and cardiac disease.

There is extensive discussion as to whether NAFLD is simply a marker or a mediator (pathogenic factor) of cardiovascular/cardiac diseases^(^^77^^)^. Underlying mechanisms are unclear due to the intricate biological interactions between NAFLD, visceral obesity and insulin resistance, all three sharing many metabolic features and risk factors. Expanded and dysfunctional visceral adipose tissue disturbs the cross-talk with the liver and possibly also the altered gut microbiota may be important^(^^77^^)^.

To date, there is no licensed (drug) treatment for NAFLD. Therefore, based on the known pathogenesis of NAFLD, several clinical trials with different nutritional supplementation and prescribed drugs have been undertaken or are currently underway. Experimental evidence has emerged about the health benefits of *n*-3 fatty acids, although current evidence is inconclusive^(^^87^^–^^89^^)^. Recent work investigating the effects of genetic variants of the patatin-like phospholipase domain-containing protein 3 gene (PNPLA3 148MM variant) supports the notion that it may be important to determine the amount of liver fat accumulation in NAFLD. This genetic variant tends to be associated with more severe liver fat accumulation and more severe NASH^(^^90^^,^^91^^)^. In a subsequent analysis of the WELCOME trial, it appeared that the PNPLA3 148MM variant that was present in about 20 % of recruited NAFLD patients influenced the effect of *n*-3 fatty acid treatment and in this *post hoc* analysis was associated with an attenuated impact of the fatty acid treatment^(^^92^^)^.

Byrne elaborated on the question of how much liver fat reduction is needed to produce a clinically relevant treatment effect and concluded it is difficult to answer this question, but probably the more liver fat reduction the better. The assessment of NASH *v*. NAFLD is currently mainly assessed by biopsy, but recent technological advancements in MR techniques^(^^93^^,^^94^^)^ may circumvent the need for biopsies to make this distinction and may help to answer the above question in the near future^(^^95^^)^.

He summarised his session by stating that fatty liver is certainly a risk factor for T2DM and is probably a risk factor for CVD. The reduction in liver fat (in people with NAFLD) has a powerful effect on T2DM, glycaemic control and hypertension. At present, it is less clear whether the presence of fatty liver adds to risk prediction for CVD over and above conventional CVD risk factors. The contribution of NAFLD to CVD risk prediction is also difficult to assess, due to extreme collinearity between metabolic CVD risk factors.

## Improving fatty liver via dietary means: the nutritionist's perspective

While Professors Byrne and Pfeiffer had already mentioned some beneficial dietary effects, Associate Professor Ulf Risérus fully focused on diet. He stated that diet-induced weight loss via energy restriction (or via physical activity; see the part of Professor Schrauwen) is the most effective way to reduce liver fat, with effects apparent already within a few days^(^^96^^,^^97^^)^. The opposite is also true. Liver fat increased with increasing energy intake after 3–4 d hyperenergetic high-fat diets^(^^98^^,^^99^^)^. At significant weight loss (>7 %) dietary composition may not influence liver fat^(^^100^^)^. When weight loss is small, however, dietary composition may influence liver fat content, although only very few studies explored effects of macronutrient composition on fatty liver in the absence of weight loss. Both low-fat and low-carbohydrate diets reduce liver fat, especially if hypoenergetic. In the short term, carbohydrate restriction seems somewhat more effective in reducing liver fat and certain metabolic risk markers than a low-fat, high-carbohydrate diet^(^^74^^,^^101^^,^^102^^)^. Risérus stressed, however, that in the low-fat diets used typically all types of fat (including PUFA) have been reduced. Moreover, long-term data are lacking to show advantages of low-carbohydrate/high-fat diets over high-carbohydrate/low-fat diets in managing NAFLD and its complications, but limited data show no difference^(^^14^^)^.

Dietary fat composition may be important in liver fat accumulation, as suggested by cross-sectional data in humans: PUFA have been inversely associated and SFA have been directly associated with liver fat (e.g. Allard *et al.*^(^^103^^)^, Musso *et al.*^(^^104^^)^, Petersson *et al.*^(^^105^^)^ and Petit *et al.*^(^^106^^)^). Furthermore, the major dietary PUFA linoleic acid (18 : 2*n*-6) has been inversely linked to T2DM risk^(^^107^^)^. SFA may induce lipogenic genes and promote liver fat as compared with PUFA^(^^107^^)^, and PUFA, but not SFA, could down-regulate lipogenic enzymes in liver, and are more readily oxidised^(^^108^^,^^109^^)^. So far, no trials comparing MUFA directly with SFA have been published, although one study in obese patients with diabetes suggests that MUFA in place of carbohydrates leads to greater reduction of liver fat^(^^13^^)^.

A recent meta-analysis^(^^110^^)^ looked specifically into the effect of *n*-3 PUFA supplementation at doses from 1 to 4 g/d for 2–24 weeks on liver fat changes, although only one study used MRS to quantify liver fat. The pooled data suggest that *n*-3 supplementation is capable of reducing liver fat, but the authors also concluded that the optimal dose is unknown and well-designed randomised controlled trials are needed. Three more recent larger controlled studies show inconsistent effects^(^^88^^,^^92^^,^^111^^)^, but these concentrated on patients with established NASH, used supplements and did not all assess compliance. Also the comparator (placebo) may explain the result. *n*-3 Fatty acids could still have preventive effects, but that requires more insight into the specific roles, modes of action, and doses of DHA and EPA^(^^112^^)^. Similar to *n*-6 PUFA, also *n*-3 PUFA such as EPA and DHA seem to down-regulate lipogenic gene expression in the liver, an effect that should be beneficial in NAFLD.

There is evidence that *n*-6 PUFA substituted for SFA could help prevent and/or treat fatty liver. Petersson *et al*.^(^^105^^)^ found that stearoyl coenzymeA desaturase-1 and insulin resistance were both independently associated with liver fat markers in elderly men. When overfeeding (+750 kcal/d; +3138 kJ/d) healthy lean subjects for 7 weeks using muffins with either SFA or *n*-6 PUFA, both groups gained a similar amount of weight (+1·6 kg), but the SFA intervention increased liver fat, while the PUFA did not^(^^15^^)^. Interestingly, the 40 g/d PUFA intervention led to an increased lean tissue mass (about 1 kg). The same research group^(^^12^^)^ also found that *n*-6 PUFA as compared with SFA reduced liver fat content significantly in abdominally obese subjects during a 10-week isoenergetic diet. Although these two trials are not directly comparable (hyper- *v*. isoenergetic), they suggest that SFA *per se* rather than SFA type is important. In one study the SFA source was mainly butter, whereas it was palm oil in the other, but in both cases these SFA diets increased liver fat compared with linoleic acid from sunflower-seed oil mainly.

While the effect of overfeeding studies with fat depends on the type of fat, also the type of carbohydrates appears to be important. High doses of sugars (e.g. sodas), and especially fructose during energy excess, could increase liver fat. For example, sugar (candy and sweet drinks) overfeeding for 3 weeks in overweight subjects increased liver fat (27 %) accompanied by increased DNL^(^^113^^)^. However, it is unclear whether increased liver fat seen after overfeeding (i.e. excess energy) with soft drinks or fructose is specific for intake of fructose or excess energy^(^^114^^,^^115^^)^.

Next to the macronutrients carbohydrates and fat, several micronutrients and phytochemicals have been tested. Supplementation with vitamins E and C have been tested in trials, especially vitamin E. Pastori *et al*.^(^^116^^)^ showed that in Italian individuals with cardiometabolic risk factors, vitamin E is significantly decreased in individuals with simple steatosis and in those with NASH. Available evidence suggests that vitamin E treatment (800–1000 IU) seems to be of benefit for improving histology and liver enzymes in NASH patients, but American guidelines for NAFLD treatment recommend vitamin E as a first-line pharmacotherapy only for non-diabetic patients with biopsy-proven NASH^(^^117^^)^. Due to safety concerns, vitamin E is not recommended in NASH patients with diabetes and high CVD risk. Studies that test vitamin E in NAFLD or as prevention are lacking^(^^117^^)^.

Cross-sectional observations have been reported between vitamin D serum levels, NAFLD risk factors, and NAFLD severity^(^^118^^)^. Whether vitamin D supplementation could be a new therapeutic option in NASH needs to be tested in trials^(^^118^^)^.

Resveratrol has been shown to lower liver fat in one study in obese subjects^(^^25^^)^, but did not lower liver fat in those with NAFLD^(^^119^^)^. Although clinical evidence is lacking, dietary cholesterol may increase liver fat^(^^120^^)^, whereas the potential beneficial effects of choline, betaine, l-carnitine, caffeine/coffee and tea should be tested.

Dr Risérus concluded that, although dietary interventions can lower liver fat, it is currently unknown how much of liver fat reduction/prevention is considered relevant in view of health and disease prevention or treatment.

## Outcome of the group discussions: answers to key questions

### Q1. Is fatty liver (accepted as) an independent risk factor for type 2 diabetes mellitus?

In subgroup discussions, the workshop participants reformulated the question into ‘Is fatty liver (accepted as) a causal risk factor for T2DM?’. The workshop participants concluded that there is no doubt about an existing robust association between fatty liver and risk of T2DM. There is also a plausible mechanism linking liver fat to glycaemic control (i.e. liver fat accumulation may reduce hepatic insulin sensitivity, thereby leading to less suppression of hepatic glucose output). However, it was also recognised that residual confounding may explain part of the observed association between liver fat and risk of T2DM; for example, fatty liver is associated with obesity, which is a risk factor for T2DM *per se*, and the ‘effects’ of liver fat, other fat ectopic depots and insulin resistance on T2DM cannot be readily disentangled. Interventions specifically targeting liver fat without affecting other risk factor for T2DM do not exist and, therefore, there is no evidence from randomised controlled trials that can prove causality. Nevertheless, the (possibly confounding) role of other fat depots in the relation between fatty liver and T2DM should also be further explored, preferably based on the currently available sensitive MRS methods, rather than less accurate liver fat measures.

However, it was also noted that liver fat is already seen by many medical experts and health care professionals as a relevant physiological target and that the ultimate ‘proof of causality’ of fatty liver in T2DM or CVD development may not be required for fatty liver being a relevant target for dietary improvements.

### Q2. Is fatty liver (accepted as) an independent risk factor for cardiovascular disease?

As for Q1, the workshop participants involved in this discussion reformulated the question into ‘Is fatty liver (accepted as) a causal risk factor for CVD?’. It seems obvious that if Q1 is answered affirmatively (fatty liver causes diabetes), then it will also cause CVD. So, a subsequent question is whether fatty liver would cause CVD independent of its relation with insulin resistance and diabetes. The participants concluded that the overall answer is ‘possibly’. Key arguments given for the low level of certainty were the limited number of studies, a stronger relation of liver fat to insulin sensitivity than to other CVD markers, the lack of mechanistic understanding beyond mechanisms related to and independent of T2DM, and the weak association of fatty liver to CVD risk, with a high susceptibility to confounding. The latter might explain why there seems to be no additional risk of CVD explained by liver fat, over and above other established markers.

Key gaps for further research were identified. The role of the inflammatory state of liver fat (NASH *v*. NAFLD) needs to be further studied, as that may be the route for an effect of fatty liver on CVD not mediated through diabetes. Although it has been shown that CVD risk is higher in NASH than in NAFLD, liver fat may still be associated with CVD risk when it is not inflamed. More mechanistic understanding is needed to understand and further explore the link between liver fat and CVD risk. Postprandial effects (on lipids and insulin) and the effects of different dietary fats on the liver fat content (amount and type) could provide a mechanistic basis for the association of liver fat with CVD. Finally, the genetic determinants of these metabolic processes, and their link to liver fat, should be further explored.

In conclusion, liver fat may be seen as an ‘aggregated’ marker for metabolic health in general. Based on limited data, it is currently not certain whether liver fat is a (causal) risk factor for CVD.

### Q3. Can the amount of liver fat be measured non-invasively, reliably and validly?

The workshop participants concluded that MRS can be used to study the effect of dietary interventions in a research context, i.e. in relatively small groups. This method is non-invasive, reliable, valid, accurate and sensitive and can now be regarded as the ‘gold standard’ of non-invasive methods. As the MRS fat signal is normalised to the water signal, care should be taken that the water content remains unchanged, which can be monitored by MRI. MRI can also be used to determine unequally distributed (focal) liver. When the amount of liver fat needs to be determined in subjects with low amounts of liver fat, e.g. most healthy subjects, the MRS protocol needs optimisation (e.g. longer acquisition and advanced post-processing). MRS measurements last about 20–30 min (in addition to the preparation time), while MRI measurements last about 10 min. Therefore, MRI can be the method of choice to accommodate more subjects, but at the expense of sensitivity.

In the clinical setting ultrasonography is used to measure hepatic fat. However, this method is only able to detect severe steatosis and is problematic in obese subjects. Therefore, this method is less suited when small changes are expected. In larger population studies, liver enzymes are used as a proxy for liver fat, but this method is even less specific and reliable, and may have led to incorrect conclusions with regard to the relationship between (prevalence of) liver fat, other metabolic derangements, and T2DM and CVD risk. Nevertheless, measuring enzymes that reflect liver function can be of relevance for selecting subjects at risk. In that case, further investigation of liver fat content in these subjects by imaging is needed to confirm a fatty liver.

MRS methodology is advancing rapidly. MRS can measure not only the amount of hepatic fat, but also the different fat types (SFA, MUFA, PUFA). However, this requires more measurement time (40–80 min) and more post-processing time. Current methodology allows the measurement of hepatic fat types, but its reliability and sensitivity still need to be determined. Kinetic responses of fat after a meal can be measured using labelled fat. All these methods can facilitate mechanistic understanding and thus can help to investigate causality between changes in liver fat and CVD and T2DM risk.

### Q4. What amount of liver fat reduction/prevention is considered relevant in view of health and disease prevention?

The workshop participants concluded that there are no clear and sufficient data to make the quantitative assessment that would allow for a confident estimate of this. Probably the more reduction in liver fat the better, but it depends on the genotype (e.g. the PNPLA3 148MM variant) and the stage of liver fat accumulation. Better phenotyping of the types of liver fat (e.g. saturated *v*. polyunsaturated fats) and the inflammatory state of the liver may be helpful in predicting the impact of liver fat reductions. In studies that show beneficial effects of dietary interventions on established metabolic risk factors, the accompanying changes in liver fat seem to suggest that at least 20 % reduction of liver fat in subjects with at least 5 % liver fat is probably necessary to achieve clear metabolic benefits. However, this statement was not supported by all participants and clearly more data are needed to confirm that.

It was noted that there is a widely held belief that prevention or reduction of liver fat would be a physiologically beneficial effect. However, to judge ‘relevance’, there is a need to be clear on which adverse health risks precisely will be reduced.

### Q5. Which dietary approaches are effective in reducing or preventing fatty liver?

The workshop participants rated the strength of evidence and concluded that the only dietary approaches that were proven (strong evidence) to affect liver fat were hypoenergetic diets and (under limited conditions) vitamin E administration. The effect size of hypoenergetic diets is largely a function of duration × energy deficit. Limited data suggest the carbohydrate *v*. fat ratio does not have much differential effect on liver fat under hypoenergetic conditions. Vitamin E has only been shown effective as an intervention in the specific situation of NASH without T2DM. Most trials have focused on the treatment effects of vitamin E, and some participants questioned whether vitamin E (and also vitamin C) might be (better) seen as preventive.

Strength of evidence for a beneficial effect of exchange of SFA by PUFA was rated as ‘probable’. The *n*-3 PUFA were mentioned as well, but to judge their efficacy, larger randomised controlled trials are needed that also take account of PNPLA3 genotype at randomisation.

Dietary approaches for which the strength of evidence was rated as ‘possible’ were the beneficial exchange of carbohydrates by MUFA, and the carbohydrate type/source (including low GI/more fibre) when in energy balance.

The evidence for resveratrol was seen as mixed. The view was there are probably small positive effects, but inconsistent efficacy may reflect differing test conditions. It was discussed whether other flavonoids could be efficacious.

Dietary approaches rated as ‘proven ineffective’ were oat fibre and coffee. Other approaches were discussed, but have been hardly studied for effects on liver fat, such as protein (content or source ((plant/animal)) or polyphenols derived from specific food sources such as tea.

## Overall conclusions

The majority of participants concluded that non-alcoholic liver fat may best be seen as an ‘aggregated’ marker for metabolic disturbances, though with a possible causal contribution of its own.

The number (and quality) of epidemiological studies or long-term intervention studies is currently insufficient to assess whether liver fat is a causal factor for T2DM or CVD, although participants were generally more convinced of a causal relationship for T2DM than for CVD. It was questioned whether a direct link of liver fat to CVD risk is relevant, especially when liver fat is associated with T2DM risk, which is a risk factor of CVD.

More robust evidence from epidemiological studies and clear mechanistic understanding are needed, e.g. on the role of the inflammatory state of the fatty liver, genetic predisposition and acute *v*. longer-term effects of dietary factors on liver fat.

MRS methodology is advancing rapidly and can measure not only the amount of hepatic fat, but is also the preferred technique for measuring changes upon interventions. Its reliability and sensitivity for measuring the fat composition of the liver (SFA *v*. PUFA) remain to be assessed. Kinetic responses of fat after a meal can be measured using labelled fat. All these methods can facilitate mechanistic understanding and thus can help to test for causality between changes in liver fat and CVD and T2DM risk.

It is still not fully established what amount of liver fat reduction/prevention is considered relevant in view of health and disease prevention.

The only dietary approaches with established effects on liver fat are a hypoenergetic diet and vitamin E, the latter only in specific circumstances. There is ‘probable’ evidence for favourable effects of replacing dietary SFA with PUFA. For *n*-3 PUFA, larger randomised controlled trials are needed. Strength of evidence for dietary approaches that were rated as ‘possible’ was the beneficial exchange of carbohydrates by MUFA, and the carbohydrate type/source (including low GI/more fibre) when in energy balance.

Dr Mela finalised the workshop and formulated the relevance for food-based approaches and for the food industry, based on the outcome of the group discussions. He concluded that:

1. It may be recommended to drop the ‘D’ (‘disease) in NAFLD. This is not a disease *per se* but a physiological target and possible disease predictor, prevalent in the general population, which can change when people change their lifestyle, including diet.
2. It is reassuring to see that the dietary approaches suggested as most likely effective for fatty liver (e.g. healthy weight, quality of fats and carbohydrates) are largely aligned with existing dietary guidance and industry targets to improve health and nutrition.
3. Robust, hypothesis-based research is needed to confirm the efficacy of specific dietary components for preventing or reducing liver fat, and to establish its place as a physiologically beneficial effect (i.e. role as a cause or marker of disease risk).
4. The ability to design efficient and decisive food-based clinical studies will benefit from the emerging methods for liver fat measurement, but greater consensus is required on the relevant effect sizes.
5. Experts from academia and food companies should work together to address the gaps identified here, as a step toward improving public health guidance and providing a basis for further innovation in health and nutrition.
